# Electrochemical Deposition of Silicon-Carbon Films: A Study on the Nucleation and Growth Mechanism

**DOI:** 10.3390/nano9121754

**Published:** 2019-12-10

**Authors:** Nina K. Plugotarenko, Tatiana N. Myasoedova, Mikhail N. Grigoryev, Tatiana S. Mikhailova

**Affiliations:** 1 Institute of Nanotechnologies, Electronics and Equipment Engineering, Southern Federal University, Chekhov str. 2, 347928 Taganrog, Russiaxelga.maks@yandex.ru (T.S.M.); 2 Joint Stock Company, Taganrog Scientific-Research Institute of Communication, 347913 Taganrog, Russia; gregoryevmikhail@mail.ru

**Keywords:** nucleation, growth, electrochemical deposition, silicon-carbon films

## Abstract

Silicon-carbon films have been deposited on silicon and Al_2_O_3_/Cr-Cu substrates, making use of the electrolysis of methanol/dimethylformamide-hexamethyldisilazane (HMDS) solutions. The electrodeposited films were characterized by Raman spectroscopy and scanning electron microscopy, respectively. Moreover, the nucleation and growth mechanism of the films were studied from the experimental current transients.

## 1. Introduction

The diamond-like carbon (DLC) films are extremely alluring for their high mechanical hardness, high electric resistivity, biocompatibility, chemical inertness, low coefficient of friction, and optical transparency in the infrared range [1,2,3]. The issue of stress and poor adhesion to the substrate in DLC films is a persistent problem that could be solved by incorporation of other elements (W, Ti, Al, Si, etc.) [4,5,6]. Therefore, the incorporation of silicon is rather promising in order to obtain amorphous silicon-carbon films.

Silicon-carbon films are very promising materials for microelectronic devices operating in aggressive environments [7]. These films are used for gas sensors, ultracapacitors, field emission devices, and other applications in aggressive environments. There are many techniques for producing these films, such as magnetron sputtering [8], ion sputtering, chemical vapor deposition, pulsed laser deposition, electrochemical deposition from molten salt, and the sol-gel method [9,10,11]. However, the applications of these techniques have been limited, owing to the sophisticated equipment and precise experimental conditions, including high vacuum and high temperature. It was experimentally shown that most materials that can be deposited from the vapor phase can also be deposited in a liquid phase using electrochemical techniques and inversely [10]. The application of the liquid deposition techniques is a good prospect due to such advantages as low consumption of energy, low deposition temperature, availability for large area deposition on complicated surfaces, and the simplicity of the setup. There are some reports that have demonstrated the possibility of the electrochemical deposition of DLC films from the organic liquids such as methanol [12], acetonitrile [13], dimethylsulfoxide [14], and lithium acetylide in dimethylsulfoxide [15], in ambient conditions. However, earlier, we reported the electrochemical deposition of silicon-carbon films from methanol/ethanol and hexamethyldisilazane (HMDS) solution [16,17]. However, in the development of the synthesis of a new material, the deposition kinetics is one of the first components to be studied in detail to ensure reproducibility. Currently, there is no information about the deposition mechanisms of silicon-carbon films from organic liquids onto different substrates.

Electrochemical methods allow setting and controlling the overpotential, control charge, current, the volume of the deposited solution, and a number of nuclei comparatively easily in the system, so they are suitable for the study of the nucleation and growth of a new phase. The analysis of potentiostatic current transients allows getting more information on the mechanism and kinetics of the electrodeposition [18].

The aim of the present study is to investigate the mechanisms of the nucleation and growth of silicon-carbon films onto silicon and Al_2_O_3_/Cr-Cu substrates through experimental potentiostatic current transients. The surface morphology, as well as structural and phase composition of the films were determined from scanning electron microscopy and Raman spectra investigations, respectively.

## 2. Materials and Methods

### 2.1. Synthesis of Silicon-Carbon Films

In this communication, the silicon-carbon films were deposited on silicon (100) (the resistivity was 4.5 Om·cm) and Al_2_O_3_ substrates with a size of 12 × 17 mm^2^. In the first step, the silicon substrate was dipped in the HF solution (≈ 15%) for a few minutes, and the conducting layer (Cr-Cu) was sputtered on the surface of the Al_2_O_3_ substrate by the magnetron technique. The substrate was mounted on the negative electrode, and graphite was mounted on the positive electrode. The distance between the substrate and the positive electrode was set to 10 mm. The deposition was done from two types of solution: (1) a methanol and HMDS solution; (2) a dimethylformamide (DMF) and HMDS solution. HMDS was dissolved in analytically pure methanol/DMF, with the volume ratio of HMDS to methanol (DMF) of 1:9. The films were deposited for 30 min. The applied potential was 180 and 500 V, for methanol-HMDS and DMF-HMDS solutions, respectively.

**Figure 1:**
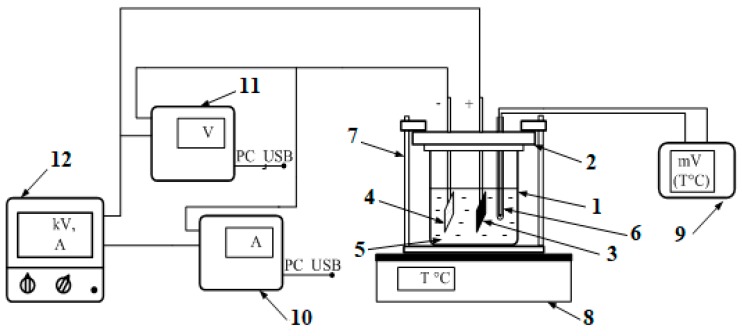
Schematic structure of electrolytic deposition system (1, glass cell; 2, dielectric cover; 3, graphite anode; 4, cathode substrate; 5, solution; 6, thermocouple; 7, clamps; 8, thermal table; 9, voltmeter of the thermocouple; 10, ammeter; 11, high-voltage voltmeter; 12, power supply).

A schematic diagram of the experimental setup is shown in Figure 1:

### 2.2. Characterization

The film morphologies were investigated using scanning electron microscopy (SEM; SEM Zeiss Merlin compact VP-60-13, Stavropol, Russia). Raman spectra were recorded at ambient temperature using a Raman Microscope, Renishaw plc (Stavropol, Russia, resolution 2 cm^−1^, 514 nm laser).

## 3. Results and Discussion

### 3.1. Characterization

**Figure 2:**
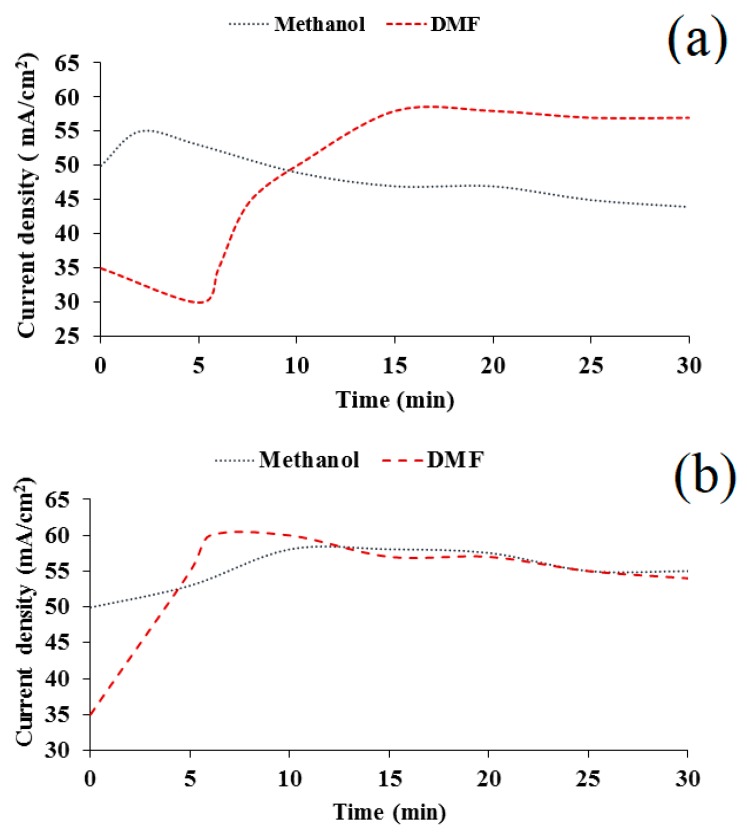
Experimental potentiostatic current transients for the deposition of silicon-carbon films on silicon (**a**) and Al_2_O_3_ (**b**) substrates.

During the deposition for a composite film from the DMF-HMDS solution, we found that the current density increased from 35 mA/cm^2^ to 54–57 mA/cm^2^ with deposition time. In the case of the methanol-HMDS solution, the current density decreased slightly from 50 mA/cm^2^ to 44 mA/cm^2^ and increased from 50 mA/cm^2^ to 55 mA/cm^2^ during the film deposition onto the silicon and Al_2_O_3_ substrate, respectively (Figure 2).

**Figure 3:**
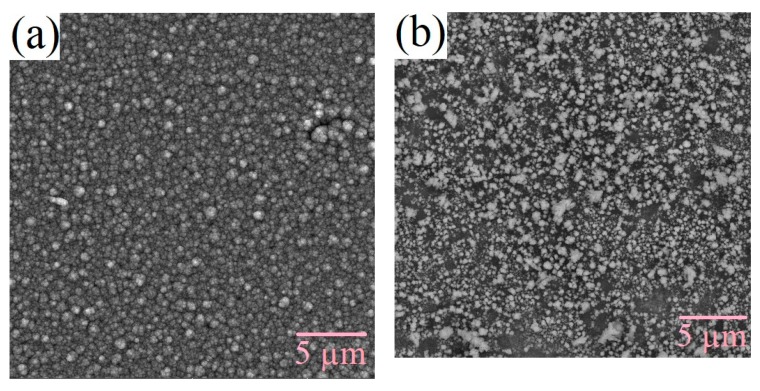
SEM micrographs of the silicon-carbon films deposited onto the silicon substrate from the methanol-hexamethyldisilazane (HMDS) (**a**) and DMF-HMDS (**b**) solutions.

**Figure 4:**
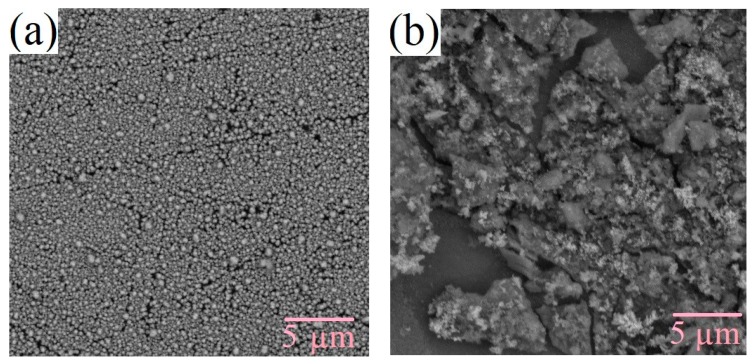
SEM micrographs of the silicon-carbon films deposited on the Al_2_O_3_ substrate from the methanol-HMDS (**a**) and DMF-HMDS (**b**) solutions.

**Figure 5:**
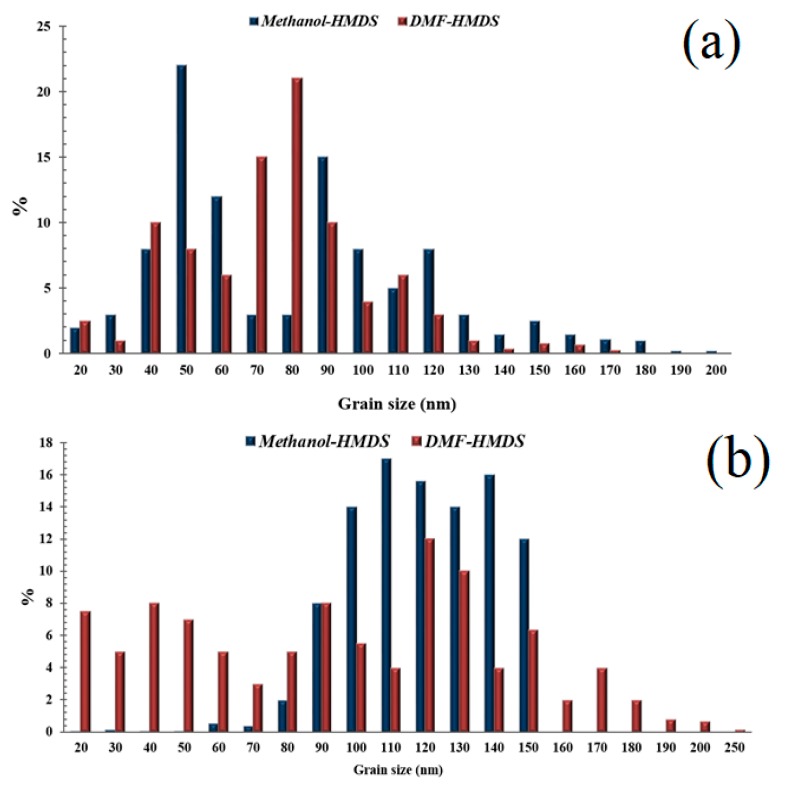
Histograms of the grain size distributions of the silicon-carbon films deposited on silicon (**a**) and Al_2_O_3_ (**b**) substrates.

The surface morphology of the films changes under varying technological conditions. The production of silicon-carbon materials is associated with thermodynamically nonequilibrium processes, which cause the formation of inhomogeneities as the films grow due to the self-organization of the structure. Figure 3 and Figure 4 shows the SEM micrographs of the deposited films. From the figures, it can be seen that films deposited from the methanol-HMDS solution and DMF-HMDS solution on the silicon substrate are composed of compact grains. The average grain size was about 90, 60, and 170 nm for the films, deposited from the methanol-HMDS on the silicon substrate, from the DMF-HMDS solution on the silicon substrate, and from the methanol-HMDS on the Al_2_O_3_ substrate, respectively. The silicon-carbon films deposited from the DMF-HMDS solution on the Al_2_O_3_ substrate characterized by a powdery structure without large grains. Therefore, the histograms of the grain size distributions were built (Figure 5).

The scatter of grain size values for the films on silicon substrates lied in the range from 20 nm to 200 nm. Grains with sizes of 50 and 80 nm predominated for the films deposited from the methanol-HMDS and DMF-HMDS solutions, respectively. For the films deposited onto the Al_2_O_3_ substrate, the histogram of the grain size values distribution was characterized by the absence of pronounced maxima. It was evident that the range of grain sizes for the films deposited from the methanol-HMDS solution was much narrower than for those deposited from the DMF-HMDS solution and was in the range of 60–150 nm.

**Figure 6:**
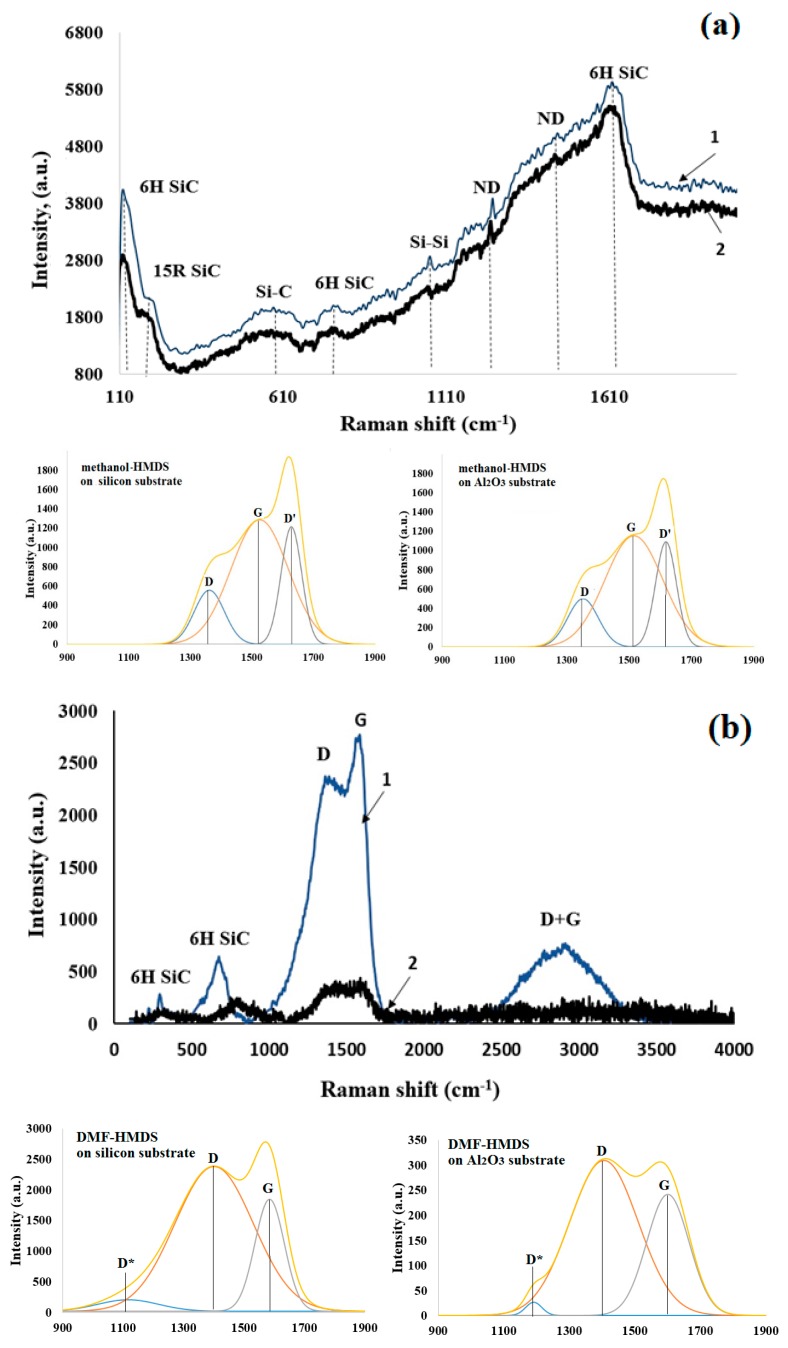
Raman spectra with the deconvolution of the D and G peaks (under the Raman spectra) of the silicon-carbon films deposited onto the silicon (1) and Al_2_O_3_ (2) substrates from the methanol-HMDS (**a**) and DMF-HMDS (**b**) solutions (D* and D’ peaks characterize disorder carbon).

The Raman spectra of the films with the deconvolution of the D and G peaks, deposited on silicon and Al_2_O_3_ substrates, are shown in Figure 6a,b, respectively.

The silicon-carbon films deposited from the methanol-HMDS solution investigated in this work were complex heterogeneous objects (Figure 6a). The Raman spectra contained the lines in the range that was characteristic of the SiC polytypes. The samples were characterized by the presence of the hexagonal 6H SiC polytype with the impurities of the rhombohedral 15R SiC phase. Furthermore, the bands attributed to the Si–C bond and nanocrystalline diamond (ND) were observed. The spectrum of the silicon-carbon film deposited on the Al_2_O_3_ substrate shifted to a lower wavenumber. The deconvolution of the Raman spectra allowed us to find out “hidden” peaks. Deconvolution was carried out on a minimum number of Gauss peak components for which their resulting curve described the experimental curve with confidence >0.99%. Therefore, in the resulting Gauss deconvolution, three peaks were observed at 1361, 1524, and 1627 cm^−1^. The peaks centered at 1361 and 1524 cm^−1^ corresponded to the conventional D and G bands. The broadening in the G band at the higher wavenumber side was due to the presence of the D’ band at 1627 cm^−1^. The appearance of the D’ peak proved that silicon-carbon films were highly defective structures [19]. The relative intensity ratio of the D peak to G peak (I_D_/I_G_) of the silicon-carbon films deposited from the methanol-HMDS solution was 1.05 for the films on both types of substrates.

Raman spectra of silicon-carbon films deposited from the DMF-HMDS solution could be characterized by the presence of the D peak and the G peak (Figure 6b). The spectrum of the silicon-carbon film on the silicon substrate was also characterized by the D + G scattering peak.

In the spectrum of the silicon-carbon film deposited on the silicon substrate, the position of the D and G peaks was 1386 and 1587 cm^−1^, respectively (Figure 6a), while the position of the D and G peaks was 1438 and 1597 cm^−1^, respectively, in the spectrum of silicon-carbon film, deposited on the Al_2_O_3_ substrate [20]. Furthermore, the G peak of the silicon-carbon film deposited on the silicon substrate shifted to a lower wavenumber, and the full width at half maximum of the G peak was also larger than that of the silicon-carbon film, deposited on the Al_2_O_3_ substrate. The high intensity of the D peak confirmed the existence of unsaturated hydrocarbons on the surface of SiC nanoparticles [21]. The bands attributed to the hexagonal 6H SiC polytype were observed.

The deconvolution of the D and G bands of the films deposited from the DMF-HMDS solution was also carried out as shown in Figure 6b. The D*, D, and G peaks were found. It should be noted that the D* peak has been found in disordered carbons. Some reports have attributed the D* peak to the sp^3^ rich phase of disordered amorphous carbons [22]. The D and G peaks were centered at 1405 (1400) cm^−1^ and 1600 (1584) cm^−1^.

Furthermore, it was seen that the relative intensity ratio of the D peak to G peak (I_D_/I_G_) of the silicon-carbon film deposited from the DMF-HMDS solution was higher than for the films deposited from the methanol-HMDS solution and reached ~1.29. The smaller ratio corresponded to smaller free carbon clusters [23].

### 3.2. Mechanism Study

The structure and morphology of silicon-carbon films depends on the nucleation and growth mechanism.

Potentiostatic transient measurement is an important method for studying the initial kinetics of electrocrystallization reactions [24,25,26].

The existing models of electrochemical deposition were based on two main ideal mechanisms for new phase nucleation on the electrode surface: instantaneous nucleation and progressive nucleation. In the case of instantaneous nucleation, all active centers are filled almost simultaneously, and further, slow growth of nuclei occurs due to the introduction of new atoms. In the presence of inhomogeneities on the surface of the substrate, germ growth first occurs at the most active centers, so with progressive nucleation, the nuclei simultaneously emerge and continue to grow. It is assumed that there is a constant supersaturation of the precursor concentration under potentiostatic conditions. Besides, both kinetic controlled and diffusion controlled growth mechanisms of a new phase on the surface are possible.

The model of 3D multiple nucleations with kinetic controlled growth was described by Isaev [18].

Instantaneous nucleation is described by:

(1) $$\frac{j}{j_{\max}}=2.34\frac{t}{t_{\max}}\omega \left(1.50\frac{t}{t_{\max}}\right)$$

where *j* is the current density, *t* is time, and *t*_max_ is the time at the maximum current.

Progressive nucleation can be expressed as:

(2) $$\frac{j}{j_{\max}}=2.25\omega_{2}\left(1.34\frac{t}{t_{\max}}\right)$$

where $\omega \left(x\right)=\exp\left(-x^{2}\right)\int_{0}^{x}\exp(\xi^{2})d\xi$ is Dawson’s integral:

(3) $$\omega_{2}\left(y\right)=\exp\left(-y^{3}\right)\overset{y}{\underset{0}{\int }}\left(y^{2}-\xi^{2}\right)\exp\left(3y\xi^{2}-2\xi^{3}\right)d\xi$$

The model of controlled nucleation was offered by Scharifker and Hills [27]. They considered the 3D nucleation model given that over time, the diffusion zones of individual nuclei overlap, which leads to a slowdown in germ growth. Instantaneous nucleation and growth are described by:

(4) $${\left(\frac{j}{j_{\max}}\right)}^{2}=\frac{1.9542}{\left(t/t_{\max}\right)}{\left\{1-\exp\left[-1.2564\left(t/t_{\max}\right)\right]\right\}}^{2}$$

Progressive nucleation can be expressed as:

(5) $${\left(\frac{j}{j_{\max}}\right)}^{2}=\frac{1.2254}{\left(t/t_{\max}\right)}{\left\{1-\exp\left[-2.3367{\left(t/t_{\max}\right)}^{2}\right]\right\}}^{2}$$

The experimental current–time transients shown in Figure 2 were analyzed using these expressions and experimentally obtained values for *j*_max_ and *t*_max_. First, the dependences of $\ln\left(1-\frac{j\sqrt{t}}{{\left(j\sqrt{t}\right)}_{\max}}\right)$ from *t* and *t*^2^ were built in order to determine instantaneous or progressive nucleation.

**Figure 7:**
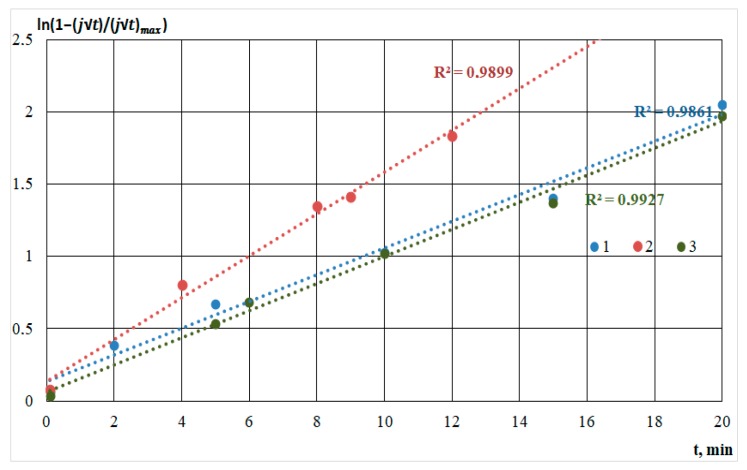
Semilogarithmic dependencies calculated from the current transients for the film deposition solution on the silicon substrate from the methanol-HMDS solution (1); on the Al_2_O_3_ substrate from the methanol-HMDS solution (2) and DMF-HMDS solution (3).

Figure 7 shows graphs of electrodeposition transients characteristic of instantaneous nucleation.

**Figure 8:**
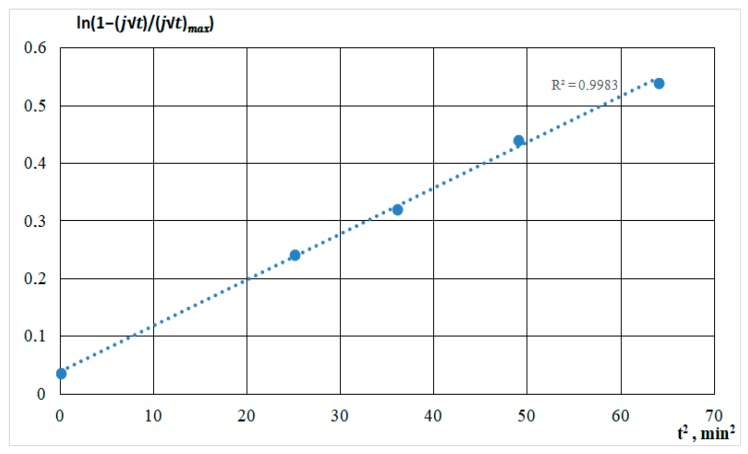
Semilogarithmic dependencies calculated from the current transients for the film deposition from the DMF-HMDS solution onto the silicon substrate.

Progressive nucleation is described by Figure 8.

As shown in the figures, for all straight lines, a high approximation confidence value was set. The comparison was based on the standard error value. Deviations from linearity were caused by concurrent processes in the solution and on the substrate: molecules’ dissociation, heating of the solution, and the formation of silicon-carbon and carbon bonds, characterizing the different growth rates.

**Figure 9:**
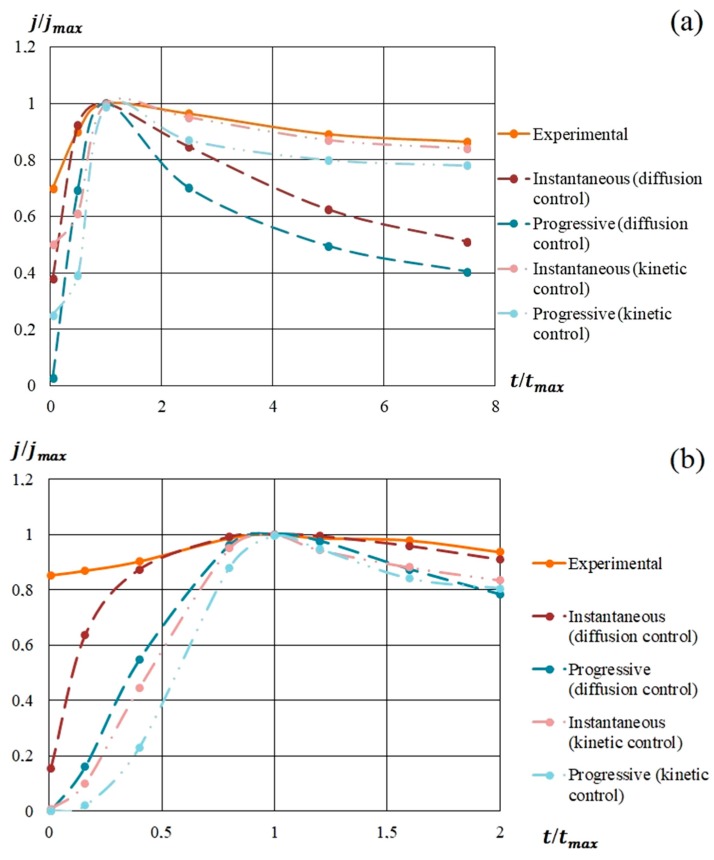

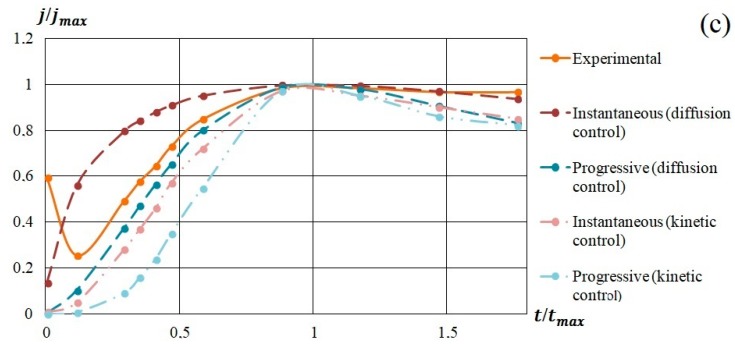

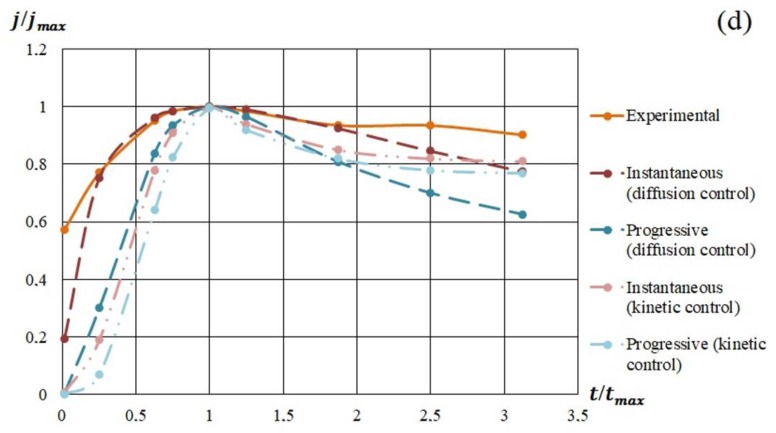
Experimental and model dependences of the current density on the deposition time of silicon-carbon films from: (**a**) methanol-HMDS solution on a silicon substrate; (**b**) methanol-HMDS solution on a Al_2_O_3_ substrate; (**c**) DMF-HMDS solution on a silicon substrate; (**d**) DMF-HMDS solution on a Al_2_O_3_ substrate.

In Figure 9, the model and experimental dependencies are presented. The analysis of the semilogarithmic and (*j/j_m_*) vs. (*t/t_m_*) dependencies showed that the mechanism of nucleation and growth of silicon-carbon films from the methanol-HMDS and DMF-HMDS solutions on the Al_2_O_3_ substrate was well described by Equation 1 for instantaneous nucleation (Figure 9b,d). The experimental current transients represented in the coordinates (*j/j_m_*) vs. (*t/t_m_*) for the deposition of silicon-carbon film from the DMF-HMDS solution on the silicon substrate demonstrated the characteristic features of the diffusion controlled growth model (Figure 9c).

The deposition of the silicon-carbon films from the methanol-HMDS solution onto the silicon substrate was characterized by the instantaneous nucleation with kinetically controlled growth (Figure 9a), while the model for instantaneous nucleation with diffusion controlled growth fit the growth mechanisms of the new phase from the methanol-HMDS and DMF-HMDS solutions on the Al_2_O_3_ substrate.

All the experimental dependences of *(j/j_m_*) vs. (*t/t_m_*) except the deposition from DMF-HMDS solution on the silicon substrate demonstrated the higher current density compared to the model for first two minutes due to the dissociation of molecules in precursors.

## 4. Conclusions

The silicon-carbon films were successfully deposited on silicon and Al_2_O_3_/Cu-Cr substrates from organic solutions. The films deposited from the methanol-HMDS solution were mostly characterized by the presence of the hexagonal 6H SiC polytype with the impurities of the rhombohedral 15R SiC phase. Raman spectra of silicon-carbon films, deposited from the DMF-HMDS, solution can be characterized by the presence of the D peak, G peak, and D + G scattering peaks of carbon and 6H SiC polytype peaks. It was shown that the nucleation and growth mechanisms depend on the nature of the solution and substrate.

## References

[1] Basa D.K., Ambrosone G., Coscia U., Setaro A. (2009). Crystallization of hydrogenated amorphous silicon-carbon films with laser and thermal annealing. Appl. Surf. Sci..

[2] Wang H., Shen M.R., Ning Z.Y., Ye C., Cao C.B., Dang H.Y., Zhu H.S. (1996). Deposition of diamond-like carbon films by electrolysis of methanol solution. Appl. Phys. Lett..

[3] Zhang Q., Wang Y., Wang W., Mitsuzak N., Chen Z. (2016). Low voltage and ambient temperature electrodeposition of uniform carbon films. Electrochem. Commun..

[4] Venkatraman C., Brodbeck C., Lei R. (1999). Tribological properties of diamond-like nanocomposite coatings at high temperatures. Surf. Coat. Technol..

[5] Ji Y., Ma M., Ji X., Xiong X., Sun X. (2018). Nickel-carbonate nanowire array: An efficient and durable electrocatalyst for water oxidation under nearly neutral conditions. Front. Chem. Sci. Eng..

[6] Ding X.Z. (2002). Structural and mechanical properties of Ti-containing diamond-like carbon films deposited by filtered cathodic vacuum arc. Thin Solid Films.

[7] Silva J.A., Quoizola S., Hernandez E., Thomas L., Massines F. (2014). Silicon carbon nitride films as passivation and antireflective coatings for silicon solar cells. Surf. Coat. Technol..

[8] Hoche H., Pusch C., Riedel R. (2010). Properties of SiCN coatings for high temperature applications—Comparison of RF-, DC- and HPPMS-sputtering. Surf. Coat. Technol..

[9] Peng Y., Zhou J. (2010). The influence of radiofrequency power on compositional, structural and optical properties of amorphous silicon. Appl. Surf. Sci..

[10] Ambrosone G., Basa D.K., Coscia U. (2010). Structural and electrical properties of nanostructured silicon carbon films. Energy Procedia.

[11] Manocha S., Ankur D., Manocha L.M. (2011). Formation of silicon carbide whiskers from organic precursors via sol-gel method. Eurasian Chem. Technol. J..

[12] Yan B., Tay B.K., Chen G., Yang S.R. (2006). Synthesis of silicon carbide nitride nanocomposite films by a simple electrochemical method. Electrochem. Commun..

[13] Guo D., Cai K., Li L.T., Zhu H.S. (2000). Preparation of hydrogenated diamond-like carbon films on conductive glass from an organic liquid using pulsed power. Chem. Phys. Lett..

[14] Jiang H.Q., Huang L.N., Zhang Z.J., Xu T., Liu W.M. (2004). Deposition of nanostructured diamond-like carbon films on al substrate by facile electrochemical route. Chem. Lett..

[15] Kulak A.I., Kokorin A.I., Meissner D., Ralchenko V.G., Vlasov I.I., Kondratyuk A.V., Kulak T.I. (2003). Electrodeposition of nanostructured diamond-like films by oxidation of lithium acetylide. Electrochem. Commun..

[16] Myasoedova T.N., Grigoryev M.N., Plugotarenko N.K., Mikhailova T.S. (2019). Fabrication of gas-sensor chips based on silicon–carbon films obtained by electrochemical deposition. Chemosensors.

[17] Grigoryev M.N., Myasoedova T.N., Mikhailova T.S. (2018). The electrochemical deposition of silicon-carbon thin films from organic solution. J. Phys. Conf. Ser..

[18] Isaev V.A., Grishenkova O.V., Zaykov Y.P. (2018). On the theory of 3D multiple nucleation with kinetic controlled growth. J. Electroanal. Chem..

[19] Kaniyoor A., Ramaprabhu S. (2012). A raman spectroscopic investigation of graphite oxide derived graphene. Aip Adv..

[20] Ferrari A.C., Robertson J. (2004). Raman spectroscopy of amorphous, nanostructured, diamond-like carbon, and nanodiamond. Philos. Trans. R. Soc. Lond. A.

[21] Mehr M., Moore D.T., Esquivel-Elizondo J.R., Nino J.C. (2015). Mechanical and thermal properties of low temperature sintered silicon carbide using a preceramic polymer as binder. J. Mater. Sci..

[22] Schwan J., Ulrich S., Batori V., Ehrhardt H. (1996). Raman spectroscopy on amorphous carbon films. J. Appl. Phys..

[23] Iijima M., Kamiya H. (2008). Surface modification of silicon carbide nanoparticles by azo radical initiators. J. Phys. Chem. C.

[24] Bijani S., Schrebler R.E., Dalchiele A., Gab M., Martínez L., Ramos-Barrado J.R. (2011). Study of the nucleation and growth mechanisms in the electrodeposition of micro- and nanostructured Cu_2_O thin films. J. Phys. Chem. C.

[25] Greef R., Peat R., Peter L.M., Pletcher D., Robinson J. (1985). Instrumental Methods in Electrochemistry.

[26] Quayum M.E., Ye S., Uosaki K. (2002). Mechanism for nucleation and growth of electrochemical palladium deposition on an Au (111) electrode. J. Electroanal. Chem..

[27] Scharifker B.R., Hills G.J. (1983). Theoretical and experimental studies of multiple nucleation. Electrochim. Acta.

